# Timing of energy intake and BMI in children: differential impacts by age and sex

**DOI:** 10.1017/S0007114522003014

**Published:** 2023-07-14

**Authors:** Sundus Mahdi, Andrew Dickerson, Gisele Infield Solar, Samantha J. Caton

**Affiliations:** 1 School of Health and Related Research, University of Sheffield, 30 Regent St, Sheffield S1 4DA, UK; 2 Sheffield Methods Institute, University of Sheffield, Interdisciplinary Centre of the Social Sciences, 219 Portobello, Sheffield S1 4DP, UK; 3 School of Medicine, University of Sheffield, Beech Hill Rd, Broomhall, Sheffield S10 2RX, UK

**Keywords:** Weight status, Energy intake, Meal timings, Childhood obesity, National Diet and Nutrition Survey

## Abstract

Body weight regulation may be influenced by the timing of food intake. The relationship between children’s BMI and their daily pattern of energy consumption was investigated using data from the UK National Diet and Nutrition Survey 2008–2019. The sample included 6281 children aged 4–18 years. Linear and logistic regression models investigated the timing of energy intake (10^3^ kJ) as a predictor of BMI (kg/m^2^) and healthy weight status. The models showed that children aged 4–10 years who consume more energy content after 20:00, in comparison with less energy content, had a significantly higher BMI (young girls: *β* = 0·159; 95 % CI 0·003, 0·315; *P* = 0·05; young boys: *β* = 0·166; 95 % CI 0·028, 0·304; *P* = 0·02). Similar findings were also present for boys aged 11–18 years (*β* = 0·091; 95 % CI 0·003, 0·180; *P* = 0·04), though logistic regression findings were contradictory (OR = 0·9566; 95 % CI 0·926, 0·989; *P =* 0·009). However, older girls who consumed more energy content in the morning had a significantly lower BMI (*β =* –0·464; 95 % CI –0·655, –0·273; *P* < 0·001) and a lower probability of non-healthy weight (OR = 0·901; 95 % CI 0·826, 0·982; *P* = 0·02). Physical activity reduced the likelihood of unhealthy weight status. The data suggest that food consumption later in the day in childhood and into adolescence may increase the risk of a higher BMI, especially for less active children. Developing guidance on appropriate meal timings and recommended energy distribution throughout the day could promote healthier lifestyles. Doing so may help increase parental awareness of timing of food intake and its potential impact on BMI.

The prevalence of overweight and obesity is considered a public health crisis by many researchers. Recent data from the UK report that overweight and obesity affect 27·7 % of children aged 4–5 years and 40·9 % of children aged 10–11 years^(1)^. A higher BMI increases the risk of non-communicable diseases in adulthood, including type 2 diabetes^(2)^, asthma^(3)^ and CVD^(4)^, as well as physical and mental health problems in childhood^(5)^. The causes of overweight and obesity are multifaceted^(6)^.

Recent evidence suggests that the timing of food intake might also affect body weight regulation^(7)^. Observational studies of adults suggested that a greater percentage of total energy intake during lunchtime may be protective against increased weight gain^(8,9)^. A cross-sectional analysis found less likelihood of overweight or obesity when energy intake exceeded 33 % of total energy intake at midday (between 11.00 and 17.00) and increased likelihood of weight gain when similar amounts were consumed during the evening (17.00–00.00)^(9)^. These findings remained even when controlling for physical activity and total daily energy intake. Similarly, a population-based cohort study in Spain investigated the extent to which energy intake throughout the day affects weight gain in adults, in which data were collected at baseline and at a 3·5 year follow-up. A higher percentage of energy intake at lunchtime was associated with lower risk of weight gain at follow-up, particularly amongst women^(8)^. Daytime consumption may regulate consumption later in the day, due to increased satiety or conscious control over food intake^(10–12)^.

Metabolic mechanisms help explain the connection between earlier meal timings and reduced BMI found within weight loss interventions. Postprandial metabolic processes, such as increased rate of gastric emptying and higher postprandial energy expenditure, are upregulated during the morning compared with the evening^(13)^. Energy intake in the evening may impair postprandial glucose and insulin tolerance, compared with morning energy intake^(14)^. In a randomised crossover trial, an association was found between eating a late lunch at 16.30 and decreased resting-energy expenditure, decreased glucose tolerance and thermic effect of food^(15)^. Likewise, individuals with obesity had less weight loss success when they consumed a late lunch (after 15.00), in comparison with those who consumed an early lunch (before 15.00), controlling for confounding factors such as total energy intake, energy expenditure and sleep duration^(16)^. Jakubowicz *et al*. investigated the distribution of energy intake throughout the day in a sample of ninety-three women with metabolic syndrome^(17)^. Participants who consumed a larger breakfast and a smaller dinner (700 and 200 calories, respectively) showed greater weight loss post-intervention than those who consumed a smaller breakfast and larger dinner (200 and 700 calories, respectively), even when total energy intake was controlled for.

The timing of the evening meal may impact on weight loss success. Women were randomised into an early evening meal group (19.00–19.30) or a late evening meal group (22.30–23.00) for 12 weeks. Those randomised into the early evening meal group had greater weight loss success and greater reductions in metabolic risk markers associated with overweight/obesity^(18)^. In addition, a recent pilot study explored the impact of time-restricted feeding over a 10-week period and its effects on body fat^(19)^. Participants in the intervention group were required to delay breakfast and advance dinner by 1·5 h each to their usual mealtimes. Daily energy intake in the intervention group was lower compared with the control group, and there was also a significant reduction in percentage body fat^(19)^.

Data from childhood observational studies have suggested that a larger energy intake in the evening may have a negative impact on weight status. An investigation of the National Health and Nutrition Examination Survey in the USA examined whether an association existed between energy consumed after 16.00 and the weight status of children aged 2–18 years. Dietary data collected through a 24-hour recall suggested that the proportion of energy consumed in the evening was positively associated with overweight amongst 6–11-year-olds, but negatively associated with body weight in 12–18-year-olds^(20)^. Similarly, Thompson and colleagues found a significant positive association between BMI and food intake between 17.00 and 06.00 amongst healthy 8–12-year-old girls^(21)^. Though these findings are not fully understood, research suggests that healthy weight preschool children have a greater tendency to consume larger breakfasts than children with overweight or obesity^(11)^. Not eating breakfast (or ‘breakfast skipping’) could potentially lead to the overconsumption of energy-dense foods later in the day and excessive energy compensation through snacking^(22)^. Previous studies have also suggested an association between meal skipping earlier in the day and an increased risk of weight gain^(23)^. In addition, a greater percentage of total daily energy intake during the evening meal was associated with increased adiposity^(24)^.

A repeated cross-sectional survey using the UK National Diet and Nutrition Survey Rolling Programme (NDNS; 2008–2012) investigated the relationship between the timing of 4–18-year-old children’s evening meal (14.00–20.00 *v*. 20.00–22.00) and weight status. No significant associations were found between evening mealtime and increased risk of being with overweight or obesity, including when participants were split by age and sex^(25)^. Although there were no statistically significant findings, this may be due to the classification of the ‘evening meal’ as the largest meal consumed between 14.00 and 22.00. In addition, only four waves of data were available for the analysis. This may have limited the number of children with overweight or obesity within the sample for any meaningful associations to present.

There are now eleven waves of NDNS data available for analysis. The inclusion of additional waves will increase the power to detect any significant associations between mealtime energy intake and weight status. In addition, more recent waves of the NDNS incorporate measures of physical activity which can then be controlled for in the analyses. Finally, incorporating the timing of *all* consumption throughout the day will provide further insights into the impact of breakfast consumption as well as evening meal consumption on child weight status. As such, the present study aims to investigate the relationship between energy intake throughout a 24-hour day and BMI/weight status in 4–18-year-old children, using data obtained from the NDNS waves 1–11 (2008–2019). Due to the differences in energy requirements, metabolic processes and circadian rhythms by age and sex^(26–28)^, this study also aims to investigate any differences in associations between timing of energy intake throughout the day and BMI/weight status by age and sex.

## Methods

### The national diet and nutrition survey

The NDNS Rolling Programme is a repeated cross-sectional survey which aims to collect nationally representative data regarding diet, nutrient intake and nutritional status in the UK population. Data were obtained from adults and children aged 1·5 years and over living in private households. Secondary analysis was undertaken of the NDNS Rolling Programme 2008–2019^(29)^ to investigate whether the timing of energy intake (kJ) throughout the day is associated with BMI and weight status. Main methods of the NDNS relevant to this study are outlined below.

### Ethics

Informed consent was obtained from participants, and anonymised data were accessed through the UK Data Archive (University of Essex, UK). For NDNS Rolling Programme 2008–2013, ethical approval was received from the Oxfordshire A Research Ethics Committee (Ref. No. 07/H0604/113)^(30)^ and from the Cambridge South NRES Committee thereafter (Ref. No. 13/EE/0016)^(31)^.

### Sample

Households were randomly selected using data from the Postcode Address File, a database of all the addresses in the UK. Typically, one adult (19 years+) and one child (1·5–18 years) would be randomly selected from a household, and for the purposes of boosting child recruitment, children were recruited from additional households. On average, 730 adults and 700 children were recruited per year between 2008 and 2019 (waves 1–11). Participants completed interviews that consisted of recording individual and household characteristics, and they recorded a food diary for three or four consecutive days (98 %+ of participants completed 4 days of food diaries). Additionally, height and weight measurements were recorded by the interviewer, and a physical activity questionnaire was undertaken from NDNS wave 6 (2014) onwards.

### Dietary data

Respondents were asked to complete a daily food diary on four consecutive days, which was a record of all the food and drink they had consumed in each 24-hour period. Respondents over the age of 12 years were asked to complete their own food diaries, otherwise a parent/carer would complete the diary with help from the child where necessary. In circumstances where the child was consuming food without the presence of the individual keeping the diary, a food and drink recording sheet was given to the carer responsible. Participants were required to provide detailed descriptions of foods consumed, as well as timing of food intake. The information was used to identify each food item and to allocate a corresponding food code using the NDNS nutrient databank as well as an appropriate portion code. In addition, data covering nutrients, such as energy (kcal), macronutrients (g) and micronutrients (mg), were also collected. A small number of children (< 2 %) only completed three food diary days; these were still retained for analysis.

### Measures

The subsections below outline the methods conducted by the authors and measures adopted for the purposes of this study. A distinction has been made where data were derived directly from the NDNS data set or produced by the authors. Methods for statistical analysis were specifically undertaken by the authors for the purposes of this research.

#### Energy intake across time

Total energy intake (converted to kilojoules, kJ) was derived by the authors for each diary day for each individual and was also categorised into one of the following time slots: 05.00 to 10.59 (morning), 11.00 to 14.59 (afternoon), 15.00 to 19.59 (evening) or 20.00 to 04.59 (night-time). Each time frame was generated as a new variable, and observations included the total energy consumption during that time frame. To retain the variability in food intake within and between individuals, data for each of the four diary days available were treated as separate observations, rather than calculating average consumption across diary days per individual. No distinction was made regarding the source of energy intake, for example from a meal, snack or beverage. Therefore, the aforementioned time frames were not categorised or referred to as mealtimes, though it would most likely include the time frame when a meal was consumed.

#### Socio-demographic variables

Child data were split into younger *v*. older categories and analysed by age (4–10 years and 11–18 years) and sex (male and female), due to the differences in food intake and recommended dietary guidelines^(26)^. We controlled for several sociodemographic variables, which were available within the data set, including equivalised household income, which takes into consideration household size and composition (three tertiles: low, mid and high), and ethnicity (White/mixed ethnic group/Black or Black British/Asian or Asian British/any other group).

#### Physical activity

Physical activity measures were available in a subsample of respondents (5–15-year-olds) recruited in years 6–11 (2014–2019) of the rolling programme. Physical activity was originally measured through a computer-assisted personal interviewing method and covered aspects of home activities, school activities and commuting. The first variable summarised whether the child met weekly physical activity recommendations for children 5–15 years old (‘low’; ‘medium’ – 60 min+ on 3–6 d or 30–59 min on all 7 d or ‘high’ – 60 min + on all 7 d). The second variable recorded the time spent doing all activities the prior week (no time/less than 1 hour/less than 3 h/less than 5 h/less than 7 h/7 h or more).

#### Anthropometric variables

A valid BMI measure was obtained from the NDNS data set. This was derived from height and weight measurements, taken by the interviewer (and which excluded unreliable measurements), and was adopted for the use within linear regression analysis. A categorical BMI measure available within the NDNS data set, which categorised children according to the UK90 children’s BMI standards through the centile method, labelled children as either healthy weight, overweight or with obesity. For the purposes of the logistic regression, we grouped children who were with overweight or obesity into a ‘non-healthy weight’ group.

### Statistical analyses

Data preparation and analysis were undertaken using STATA/se 16·1. For the purposes of this study, all data sets for waves 1–11 were merged, and data from 4- to 18-year-olds were only analysed (*n* 6281). This age group was selected as the focus of this research was on school-age children. This study did not include toddlers and infants within our investigation due to differences in dietary requirements^(26)^ and daily routines^(28)^. Sampling weights were constructed from the data set to correct for differential sampling and response rates in order to make the data representative for all children aged 4–18 years in the UK. Total kJ consumed was calculated for each of the four time periods (morning, afternoon, evening and night-time) considered per diary day. New variables were also produced reflecting the percentage of total kJ consumed over the day for each period. Descriptive statistics were generated by sex and age (4–10 years and 11–18 years) to reflect patterns of consumption during the day. For a base-case analysis, a linear regression was conducted exploring the association between BMI (kg/m^2^), age, sex, equivalised household income, ethnicity and total energy intake per day (kJ). The total energy intake was then substituted with (i) the energy consumed in the four time periods, (ii) percentages of energy consumed across the four time periods, controlling for total energy consumed (results not reported) and (iii) energy consumed over the four time periods differentiated by age and sex. The method outlined above was also repeated for a binary logistic regression exploring the relationship between ‘weight status’ (defined as being with overweight or obesity *v*. healthy weight), energy intake and the other confounders.

The above analysis was then repeated for respondents aged 5–15 years in waves 6–11 of the NDNS (2014–2019) in order to consider physical activity within the analysis. Physical activity was included in the same linear and logistic regression models as described above, to explore the impact of energy intake on BMI and weight status, conditional on physical activity.

The self-reported diary data may have been affected by potential underreporting and a lack of robustness. This was investigated by re-running the analysis on only 12- and 13-year-olds separately. These ages were chosen as they span the cut-off age between a parent/carer completing the food diary on behalf of the child, and the child completing it on their own. Due to the one-year age difference, we expected the relationship between BMI and energy intake to be very similar, and hence any observed differences could indicate the potential of measurement error in self-reporting.

## Results

### Characteristics of study sample

In total, our sample consisted of 6281 children and adolescents, of which 48 % were 4–10 years old (‘younger children’) and 52 % were 11–18 years old (‘older children’). The mean age amongst younger and older children was approximately 7·0 and 14·5 years, respectively. Around 34 %, 29 % and 24 % of the sample were in tertiles 1 (low), 2 (mid) and 3 (high) of equivalised household income, respectively. Approximately 12 % of households did not report their income to the interviewer. The sample was predominantly of White ethnicity (81·3 %), followed by Asian/Asian British (9·2 %). Table 1 presents further information on study sample characteristics.

**Table 1.** Sample characteristics: age, sex, ethnic group and household income

	4–10-year-olds		11–18-year-olds		
	Boys	Girls	Boys	Girls	Total
Share (%)	24·0	22·9	27·2	25·8	100·0
Age (years)					
Mean	6·99	6·98	14·43	14·53	10·96
sd	2·00	2·05	2·27	2·23	4·31
Ethnic group (%)					
White	81·9	79·0	81·7	82·3	81·3
Mixed ethnic group	4·3	5·0	3·0	2·8	3·7
Black/Black British	2·7	4·1	4·5	3·7	3·8
Asian/Asian British	8·8	10·3	8·9	9·0	9·2
Other	2·4	1·6	1·9	2·2	2·0
Total	100·0	100·0	100·0	100·0	100·0
Tertiles of equivalised household income (%)					
1st (low)	32·6	33·3	35·1	36·0	34·3
2nd (mid)	30·9	29·5	29·3	27·5	29·3
3rd (high)	26·4	26·5	22·5	23·0	24·5
Unreported	10·1	10·6	13·2	13·6	11·9
Total	100·0	100·0	100·0	100·0	100·0
*n* (unweighted)	1571	1440	1619	1651	6281

Source: National Diet and Nutrition Survey (NDNS) 2008–2019 – authors’ calculations.

Sample characteristics are based on weighted data.

As shown in Table 2, the average BMI of younger boys and girls was 16·8 (sd = 2·5) and 17·0 (sd = 2·8), respectively. The average BMI for older boys and girls was 21·2 (sd = 4·3) and 22·2 (sd = 4·7), respectively. The difference in BMI by sex for the older children is statistically significant (*P* < 0·001) but not for the younger children (*P* = 0·19). Approximately 74 % of younger children and 66 % of older children were healthy weight.

**Table 2.** BMI and weight status by age and sex

	4–10-year-olds		11–18-year-olds		
	Boys	Girls	Boys	Girls	Total
BMI (kg/m^2^)					
Mean	16·8	17·0	21·2	22·2	19·5
sd	2·5	2·8	4·3	4·7	4·5
Weight status (%)					
Normal-weight	73·6	75·2	66·4	65·0	69·8
Overweight	12·2	12·6	13·3	13·9	13·0
Obese	14·2	12·2	20·3	21·1	17·2
Total	100·0	100·0	100·0	100·0	100·0
*n* (unweighted)	1491	1359	1543	1585	5978

Source: National Diet and Nutrition Survey (NDNS) 2008–2019 – authors’ calculations.

Sample characteristics are based on weighted data.

### Energy intake and physical activity

Younger boys and girls consumed on average 6432 kJ and 5905 kJ per day, respectively, whereas older boys and girls consumed 7925 kJ and 6564 kJ, respectively. Energy intake varied across the four time points throughout the day and between age and sex (see Table 3). Overall, the majority of energy intake – around 40 % irrespective of age and sex – was consumed in the evening (15.00–19.59), followed by around 30–32 % in the afternoon (11.00–14·59). When split by age category, younger children consumed approximately 22 % of their energy intake during the morning (05.00–10.59) and only approximately 5 % during the night-time (20.00–04.59). On the other hand, older children consumed approximately 16 % of their total energy intake in the morning and 13 % in the night-time (see Table 3).

**Table 3.** Average daily energy intake (kJ) by age, sex and time of day

	4–10-year-olds		11–18-year-olds		
	Boys	Girls	Boys	Girls	Total
Energy intake (kJ)					
05.00–10.59	1407	1250	1301	1027	1244
11.00–14.59	2006	1890	2396	2019	2089
15.00–19.59	2648	2416	3085	258	2698
20.00–04.59	371	349	1143	931	721
Total	6432	5905	7925	6564	6752
sd	1834	1794	2926	2393	2435
Energy intake (%)					
05.00–10.59	22·2	21·5	16·8	15·7	18·9
11.00–14.59	31·5	32·4	30·1	30·9	31·2
15.00–19.59	41·0	40·4	39·5	40·0	40·2
20.00–04.59	5·4	5·6	13·6	13·3	9·7
Total	100·0	100·0	100·0	100·0	100·0
*n* (unweighted)	1571	1440	1619	1651	6281

Source: National Diet and Nutrition Survey (NDNS) 2008–2019 – authors’ calculations.

Sample characteristics are based on weighted data.

Within our sample, older girls participated in the least amount of physical activity in comparison with other groups. Specifically, 54 % took part in low amounts of weekly physical activity (defined as less than 60 min+ of physical activity on 3–6 d or less than 30–59 min of physical activity on all 7 d). Both younger and older boys engaged in the highest amount of physical activity in comparison with younger and older girls. Approximately 24 % of younger boys and 19 % of older boys engaged in a high level of physical activity (defined as 60 min + of physical activity on all 7 d). In comparison, approximately 19 % of younger girls and only 8 % of older girls engaged in high levels of physical activity. The findings also suggested that over half of younger children and 43 % of older boys participated in more than 7 h of physical activity in the past week, in comparison with only 33 % of older girls (see Table 4).

**Table 4.** Physical activity by age (5–15 years) and sex

	5–10-year-olds		11–15-year-olds		
	Boys	Girls	Boys	Girls	Total
Weekly activity level %					
Low	37·7	39·2	46·2	54·1	43·5
Medium	38·5	42·3	34·9	37·8	38·6
High	23·8	18·5	18·9	8·0	17·9
Total	100·0	100·0	100·0	100·0	100·0
Weekly activity time %					
None	4·2	3·7	11·6	14·1	7·8
Less than 1 h	4·9	3·2	3·0	8·8	4·8
1 to 3 h	11·7	14·7	17·9	18·5	15·3
3 to 5 h	12·2	15·2	13·6	15·4	14·0
5 to 7 h	13·7	14·3	10·6	10·6	12·5
More than 7 h	53·3	48·9	43·4	32·6	45·5
Total	100·0	100·0	100·0	100·0	100·0
*n* (unweighted)	676	593	463	477	2209

Source: National Diet and Nutrition Survey (NDNS) 2014–2019 – authors’ calculations.

Sample characteristics are based on weighted data. The activity-level variable is recorded for waves 6–11 (2014–2019) only and for children aged 5–15 years. See text for details.

### Energy intake as a predictor of BMI

Multiple linear regressions were carried out to investigate the conditional relationship between BMI and energy content consumed throughout the day while controlling for total daily energy intake (in 10^3^ kJ), equivalised household income and ethnicity. The results in Table 5 model 1 indicate that, in aggregate, BMI is unrelated to total energy consumption once conditioning on age, sex, income and ethnicity. However, when the timing of energy intake is considered as in Table 5 model 2, a negative association was found between morning energy intake (05.00–10.59) and BMI (*β* = –0·1781; 95 % CI –0·262, –0·094; *P* < 0·001), and a positive association was present between night-time (20.00–04.49) energy intake and BMI (*β* = 0·0875; 95 % CI 0·017, 0·158; *P* = 0·01). Across the four time periods (morning: afternoon: evening: night-time), energy intake is approximately distributed as 20 %: 30 %: 40 %: 10 % (see Table 3).

**Table 5.** Relationship between BMI and timing of energy intake

			Model 3			
Dependent var:	Model 1	Model 2	95 % CI			
BMI value (kg/m^2^)	95 % CI	95 % CI	Young boy aged 4–10 years	Young girl aged 4–10 years	Older boy aged 11–18 years	Older girl aged 11–18 years
Total daily intake	0·0075					
(kJ × 10^3^)	–0·028,0·043					
	*P =* 0·68					
Intake 05.00–10.59		–0·1781	–0·0538	–0·0460	–0·0897	–0·4639
(kJ × 10^3^)		–0·262,–0·094	–0·190,0·082	–0·213,0·121	–0·230,0·051	–0·655,–0·273
		*P* < 0·001	*P =* 0·44	*P =* 0·59	*P =* 0·21	*P* < 0·001
Intake 11.00–14.59		0·0140	0·0416	0·1154	0·0420	–0·0846
(kJ × 10^3^)		–0·042,0·070	–0·043,0·127	0·019,0·212	–0·048,0·132	–0·208,0·038
		*P =* 0·62	*P =* 0·34	*P =* 0·02	*P =* 0·36	*P =* 0·18
Intake 15.00–19.59		0·0301	0·0607	0·1443	0·0045	–0·0170
(kJ × 10^3^)		–0·013,0·074	–0·013,0·135	0·059,0·230	–0·071,0·080	–0·109,0·075
		*P =* 0·17	*P =* 0·11	*P =* 0·001	*P =* 0·91	*P =* 0·72
Intake 20.00–04.59		0·0875	0·1660	0·1589	0·0914	0·0323
(kJ × 10^3^)		0·017,0·158	0·028,0·304	0·003,0·315	0·003,0·180	–0·123,0·188
		*P =* 0·01	*P =* 0·02	*P =* 0·05	*P =* 0·04	*P =* 0·68
Young boy 4–10 years	base	base	base			
Young girl 4–10 years	0·1669	0·1523	–0·1703			
	–0·036,0·370	–0·050,0·355	–0·768,0·427			
	*P =* 0·11	*P =* 0·14	*P =* 0·58			
Older boy 11–18 years	4·4030	4·3038	4·5392			
	4·139,4·667	4·036,4·572	3·870,5·209			
	*P* < 0·001	*P* < 0·001	*P* < 0·001			
Older girl 11–18 years	5·3346	5·2183	6·2161			
	5·068,5·601	4·949,5·487	5·506,6·926			
	*P* < 0·001	*P* < 0·001	*P* < 0·001			
Constant	17·337	17·477	17·144			
	17·04,17·64	17·17,17·78	16·70,17·58			
	*P* < 0·001	*P* < 0·001	*P* < 0·001			
Ethnicity (5 groups)	✓	✓	✓			
HH income (4 bands)	✓	✓	✓			
N	23 796	23 796	23 796			
R^2^	0·302	0·303	0·305			

Source: National Diet and Nutrition Survey (NDNS) 2008–2019 – authors’ calculations.

Dependent variable is BMI (kg/m^2^). 95 % CI in [brackets]; *P* values in (parentheses). All specifications include ethnicity (five categories) and household income tertiles (four categories including non-reported).

When distinguishing by age and sex as in Table 5 model 3, older girls who consume more in the morning period having significantly lower BMI than their counterparts who consume less during this period (*β* = –0·4639; 95 % CI –0·655, –0·273; *P* < 0·001). On the other hand, young girls who consume more during the afternoon (*β* = 0·1154; 95 % CI 0·019, 0·212; *P =* 0·02), evening (*β* = 0·1443; 95 % CI 0·059, 0·230; *P =* 0·001) and night-time (*β* = 0·1589; 95 % CI 0·003, 0·315; *P =* 0·05) are found to have a higher BMI relative to otherwise similar younger girls who consume less during these time periods. Amongst boys, higher energy intake was only significantly associated with BMI during night-time energy intake (20.00–04.59); for younger and older boys, respectively, BMI is 0·1660 higher (95 % CI 0·028, 0·304; *P =* 0·02) and 0·0914 higher (95 % CI 0·003, 0·180; *P =* 0·04) for every additional 1000 kJ consumed after 20.00.

When physical activity was controlled for within a separate regression model restricted to the sample of 5–15-year-olds from NDNS waves 6–11 for whom physical activity information is available, all BMI-energy intake associations were no longer statistically significantly different from zero as shown in Table 6. On the other hand, children who engaged in high amounts of weekly physical activity (60 mins+ on all seven days of the week) had a significantly lower BMI than those who engaged in low amounts of weekly physical activity (*β*= –0·58; 95 % CI –1·024, –0·135; *P =* 0·01) and an impact of around half of this magnitude for those engaged in medium levels of physical activity.

**Table 6.** Relationship between BMI, timing of energy intake and physical activity for 5–15-year-olds

			Model 3			
Dependent var:	Model 1	Model 2	95 % CI			
BMI value (kg/m^2^)	95 % CI	95 % CI	Young boy aged 4–10 years	Young girl aged 4–10 years	Older boy aged 11–18 years	Older girl aged 11–18 years
Total daily intake	–0·0035					
(kJ × 10^3^)	–0·061,0·054					
	*P =* 0·90					
Intake 05.00–10.59		–0·1224	–0·1107	–0·0995	–0·0171	–0·2783
(kJ × 10^3^)		–0·270,0·025	–0·316,0·095	–0·365,0·166	–0·327,0·293	–0·625,0·069
		*P =* 0·10	*P =* 0·29	*P =* 0·46	*P =* 0·91	*P =* 0·12
Intake 11.00–14.59		0·0397	0·1149	0·1423	0·0498	–0·0898
(kJ × 10^3^)		–0·059,0·139	–0·028,0·258	–0·019,0·304	–0·141,0·241	–0·318,0·139
		*P =* 0·43	*P =* 0·12	*P =* 0·08	*P =* 0·61	*P =* 0·44
Intake 15.00–19.59		0·0021	0·0187	–0·0180	0·0236	–0·0290
(kJ × 10^3^)		–0·072,0·076	–0·087,0·125	–0·152,0·116	–0·127,0·174	–0·211,0·153
		*P =* 0·96	*P =* 0·73	*P =* 0·79	*P =* 0·76	*P =* 0·75
Intake 20.00–04.59		0·0307	0·1624	–0·0914	0·009	0·0395
(kJ × 10^3^)		–0·092,0·153	–0·046,0·371	–0·332,0·149	–0·205,0·223	–0·230,0·309
		*P =* 0·62	*P =* 0·13	*P =* 0·46	*P =* 0·93	*P =* 0·77
Young boy 4–10 years	base	base	base			
Young girl 4–10 years	0·0451	0·0363	0·1655			
	–0·282,0·372	–0·291,0·363	–0·799,1·130			
	*P =* 0·79	*P =* 0·83	*P =* 0·74			
Older boy 11–18 years	3·4233	3·3913	3·4421			
	2·960,3·886	2·928,3·855	2·333,4·551			
	*P* < 0·001	*P* < 0·001	*P* < 0·001			
Older girl 11–18 years	4·2500	4·2074	4·9886			
	3·777,4·723	3·731,4·684	3·816,6·161			
	*P* < 0·001	*P* < 0·001	*P* < 0·001			
Physical activity: Low	base	base	base			
Physical activity:	–0·2595	–0·2560	–0·2599			
Medium	–0·606,0·087	–0·602,0·090	–0·605,0·085			
	*P =* 0·14	*P =* 0·15	*P =* 0·14			
Physical activity:	–0·5793	–0·5775	–0·5763			
High	–1·024,–0·135	–1·022,–0·133	–1·021,–0·132			
	*P =* 0·01	*P =* 0·01	*P =* 0·01			
Constant	17·706	17·748	17·486			
	17·17,18·24	17·20,18·30	16·78,18·19			
	*P* < 0·001	*P* < 0·001	*P* < 0·001			
Ethnicity (5 groups)	✓	✓	✓			
HH income (4 bands)	✓	✓	✓			
N	8290	8290	8290			
R^2^	0·237	0·238	0·239			

Source: National Diet and Nutrition Survey (NDNS) 2014–2019 – authors’ calculations.

See Table 5. The physical activity level variable is recorded for waves 6–11 (2014–2019) only and for children aged 5–15 years. See text for details.

### Energy intake as a predictor of weight status

A binary weight status outcome was derived, which grouped individuals into either healthy weight or with overweight or obesity according to their BMI and the UK90 benchmarks. Based on their self-reported food diary data, individuals who are with overweight or obesity had, on average, a slightly *lower* daily energy intake than those with healthy weight (average difference = –61 kJ, 95 % CI –128·0, 6·0; *P =* 0·08). A logistic regression was conducted to predict weight status whilst controlling for equivalised household income and ethnicity. As shown in online Supplementary Table S1, in aggregate (Model 1), the results indicated that the odds of being with overweight or obesity is slightly *lower* the higher is the total energy intake (OR = 0·9780; 95 % CI 0·961, 0·996; *P =* 0·02). When considering time-of-day (Model 2), the results indicate that higher energy intake in the morning (05.00–10.59) or night-time (20.00–04.59) was significantly associated with a lower odds of being with overweight or obesity (OR = 0·9588; 95 % CI 0·917, 1·003; *P =* 0·07; and OR = 0·9566; 95 % CI 0·926, 0·989; *P =* 0·009, respectively). Model 3, which distinguishes energy intake by age and sex, shows that these results are driven by the outcomes for the older children, and particularly older girls.

Online Supplementary Table S2 replicates the analysis in Table S1 for the subsample of 5–15-year-olds for whom information on physical activity is available. In comparison with low levels of physical activity, the impact of high and medium levels to reduce the likelihood of children being with overweight or obesity was not statistically significant at conventional levels.

## Discussion

This study explored the timing of energy intake throughout the day on BMI in 4–18-year-olds using data from all eleven waves (2008–2019) of the NDNS. Results demonstrated that time of day energy intake was significantly associated with BMI and weight status conditional on ethnicity and household income. Associations varied by age and sex. When considering physical activity, the relationships between BMI and timing of consumption were weaker. For all statistically significant findings, the effects are small in magnitude.

Energy intake for the sample population shows a gradual increase in the proportion of daily energy intake throughout the day. Energy intake was found to be the lowest during the night-time (20.00 – 04.59) and highest during the evening (15.00 – 19.59), which aligns with previously reported data from the 1946 British Birth Cohort^(32)^. In addition, children consumed 13 % of their energy intake during the night-time period (20.00–04.59) in comparison with young children who consumed just 5 % in this period. Public Health England have previously advised, as part of their One You campaign for adults, a daily energy distribution of 400:600:600 calories for breakfast, lunch and dinner, respectively. The remainder of recommended energy content are to come from drinks and snacks^(33)^. In the current research, therefore, 4–18-year-olds may be under-consuming during the breakfast period and over-consuming during dinner. This is considerably more so amongst older children who typically consume 50 % of their energy intake in the evening and night-time periods. If night-time energy intake was replaced by a larger energy intake in the morning (e.g. 30 %: 30 %: 40 %: 0 % distribution of energy intake across the four time periods), then, *ceteris paribus*, BMI would be approximately 0·2 (∼1 %) lower than the average BMI of 20.

Greater energy intake in the morning was predictive of lower BMI and healthy weight status. This association was particularly the case for older girls, although only remains weakly significant (*P =* 0·09) for weight status after adjusting for physical activity. Older girls were more sedentary compared with other children and adolescents, and so greater energy consumption in the morning may upregulate metabolic processes. This is evidenced within chrono-nutrition studies; 24-hour circadian rhythms and clock genes influence metabolism processes, resulting in ideal circadian timings for when individuals should eat^(34)^. In addition, a randomised cross-over study demonstrated that postprandial metabolic processes have shown higher energy expenditure (resting metabolic rate) in the morning compared with the evening^(35)^. Research has also suggested protective factors against overweight of earlier, rather than later, food intake throughout the day^(8,17)^, with more recent evidence highlighting improved glycaemic control with increased breakfast consumption in children^(36)^.

Consuming a larger breakfast may suppress appetite and improve satiety and diet quality^(10)^. This may lead to reductions in snacking or eating energy-dense foods later in the day^(11)^. It has been previously suggested that those who consume more during breakfast are more likely to have a lower BMI^(37,38)^. Similarly, literature on breakfast skipping has evidenced a 44 % increased risk of obesity in children and adolescents^(39)^, which can be moderated through daily moderate-to-vigorous physical activity^(40)^. It has been reported within a recent review that food ingestion in the morning acts as a zeitgeber for the peripheral clock system, therefore causing misalignment of clock gene expression involved in metabolic pathways^(41,42)^. On the other hand, there was little evidence to suggest that breakfast consumption had an impact on basal metabolism in a review of studies on breakfast consumption and energy balance in adults^(43)^. The variability in findings within the literature indicates a need to conduct more rigorous research in this area.

Greater energy intake during all times of the day, excluding the morning, was associated with a greater BMI amongst younger girls. These results support existing research; a longitudinal cohort-based study investigated the association between time-of-day energy intake on BMI. Results suggested that higher lunch (11.00–14.30) and supper (after 21.30) intake, or higher mid-afternoon intake (14.30–19.00), at 4 years predicted increased BMI at 7 years. However, when controlling for physical activity, associations were no longer present within this current study. Given that almost 50 % of younger girls participated in more than 7 h of physical activity in the previous week, it is possible that physical activity moderated the relationship between energy intake and BMI, leading to a healthy energy balance^(44,45)^.

Night-time energy intake (20.00–04.59) was found to be positively associated with greater BMI amongst older boys and younger children. Given that, typically, young children have earlier bed times than older children, and evening energy intake could lead to energy storage due to declines in energy metabolism^(35)^. Alternatively, late sleep timing has been found to be associated with greater energy intake in the evening amongst 9–15-year-olds^(46)^ with recent research suggesting associations between later sleep times, lower vegetable intake and increased intake of processed and fried foods in preschool children^(47)^. Research investigating the effects of energy intake at different times of the day have also suggested that individuals consuming a high calorie dinner had higher levels of glucose and insulin values in comparison with those that consumed a low-energy dinner. This supports the notion that insulin sensitivity and glucose tolerance decrease as the day progresses, which may contribute to weight gain amongst night eaters^(17)^. Furthermore, a recent study investigating the impact of early (19.00–19.30) *v*. late (22.30–23.00) evening mealtimes on weight loss found greater weight loss success amongst women within the early evening meal group^(18)^. This further reiterates the importance of mealtime consumption and its effects on weight regulation. Despite the above, associations between night-time energy intake and BMI became statistically non-significant within our analysis once controlling for physical activity. Our analysis suggested that the majority of younger children and older boys engaged in physical activity. Given that physical activity was found to be associated with lower BMI, as has been confirmed by previous research^(48)^, it is possible that the association between night-time energy intake and BMI was moderated by physical activity. Since physical activity leads to increased energy expenditure, this could help to maintain energy balance and prevent weight gain^(49)^.

Contrary findings were present when BMI was treated as a categorical variable (i.e. weight status). Results indicated that greater night-time (20.00–04.59) energy consumption was significantly associated with a decreased likelihood of overweight or obesity amongst older boys. However, this association was lost after controlling for physical activity. Similar results were identified in analyses involving the National Health and Nutrition Examination Survey, a nationally representative sample of 2–18-year-olds in the USA. Amongst 6–11-year-olds, a positive association between evening mealtime energy intake and weight status was identified. On the other hand, 12–18-year-olds who were overweight were found to consume less later in the day^(20)^. Within our analysis, it was assumed that intake between 20.00–04.59 would most likely consist of snacks rather than a meal due to being unconventional eating hours and contributing the least towards total daily energy intake. Keast and colleagues looked to examine the relationship between snacking and weight status^(50)^. Their results indicated that greater snacking was associated with lower BMI. On the other hand, a meta-analysis investigating the association between energy intake at dinner time and weight found no association between these variables^(51)^, whilst others lost any significant association when only ‘true-reporters’ (those who accurately reported their food intake) were included within the analysis^(9)^. One potential mechanism could be the assumption that higher night-time energy intake leads to decreased energy intake the next morning or day. However, available evidence does not support this hypothesis^(52)^ and deemed it unlikely^(9)^, nor were the interrelationships between energy intake and eating occasions investigated within this study to explore this mechanism further.

A variety of significant interrelationships between time-of-day energy intake and child weight have been observed within this study. However, the associations are not causal, and the interpretation of results has proven to be challenging. The results of the logistic regression exploring night-time energy consumption and weight status are difficult to explain, given the significant direction of the association, and the contradictory results from the BMI regression results. However, the observed associations could be related to lifestyle factors that have not been considered within the analysis, such as overnight fast duration and snack content. It has been suggested that adults who had a medium or long overnight fast had a lower BMI in comparison with those with a shorter overnight fast, whilst controlling for total energy intake^(53)^. Other research within preschool aged children has similarly suggested that shorter nocturnal sleep durations (which may suggest a shorter overnight fast) among children sleeping after 21.00 was associated with a higher BMI^(54)^. Unfortunately, the duration between the last eating occasion and the first eating occasion of the next day was not considered within our analysis. Although evidence is available to suggest greater likelihood of consuming energy-dense foods in the evening and night time hours^(46)^, we did not investigate which foods were being consumed at different timepoints throughout the day within our sample. Therefore, we cannot infer whether foods consumed at night-time were energy-dense foods, or satiety-promoting foods such as fruit, which could reduce obesity risk^(55)^.

The role of sleep duration and the time between last eating occasion and bedtime should be considered as covariates within future analyses. A recent randomised controlled trial investigated the relationship between sleep duration, bedtime and obesity risk in 8–11-year-olds^(56)^. The results demonstrated that children who had a later bedtime consumed more energy after their evening meal compared with children who had an earlier bedtime. In addition, shorter sleep duration was associated with a higher standardised BMI (zBMI) and a later last meal of the day. It has also been suggested that insufficient night time sleep in preschoolers could interfere with biological processes affecting appetite and metabolism^(54)^, with meta-analysis outputs suggesting a 54 % increased risk of obesity^(57)^. Emotional overeating and increased calorie consumption in the absence of hunger has also been found to be significantly associated with poor child sleep health^(58)^. Chronotype, an individual’s preference for activity/rest in the morning/evening^(59)^, may also be a modifier of the relationship between energy intake and weight status^(60)^. It has been found that those who consumed a higher percentage of energy intake at breakfast within 2 h of waking are less likely to be overweight, only if they had an earlier chronotype. Similarly, a greater percentage of energy intake at night, within 2 h before bedtime, was associated with a greater odds of being overweight amongst later chronotypes^(60)^.

Given the use of self-reported dietary data, which could lead to underreporting, the results should be interpreted with caution. Our data suggested that those with overweight or obesity had, on average, a lower daily energy intake than those with a healthy weight. This was also supported by logistic regression data which indicated that those who consume greater energy have a reduced likelihood of being with overweight or obesity. Previous research has suggested that underreporting of food diary data is more likely for those with overweight or obesity than those who are healthy weight^(61,62)^. This may also help explain the contradictory findings outlined above between linear and logistic regression models. Similarly, data on physical activity was also self-reported which could lead to bias in estimates. Although data did not suggest any unusual findings pertaining to physical activity and BMI, previous research has suggested that participants have a tendency to underreport light and moderate intensities of physical activity, and overreport vigorous intensities when compared with objective measures (e.g. SenseWear armband). Positive correlations between self-reported and objective data was impaired by BMI, mostly within those with a higher BMI^(63)^. Similarly, other research has suggested overestimation of physical activity amongst the most inactive in a sample of adolescents with obesity^(64)^.

Several additional analyses were conducted to confirm the robustness of the findings. Given that parents/carers of children 12 years and younger completed the food diaries on behalf of the child, dietary reports for 12- and 13-year-olds were compared in order to investigate any potential self-report bias (for example, due to parents not being fully aware of everything their children consume). Similar to previous research investigating differences in reported dietary intake in child-parent dyads^(65)^, there were no significant differences in child and adult reports of child dietary intake. Furthermore, the estimated relationships between BMI and energy intake for 12-year-olds were very similar to those for 13-year-olds (results not reported).

It is important to note that our analysis incorporated eleven waves of NDNS data. Not only does this increase statistical power due to increased sample size, but it also provided an opportunity to incorporate physical activity within the analysis due to its availability in waves 6 onwards. These features of our analysis are an advance on similar research conducted on the NDNS exploring the impact of mealtimes, or timing of energy intake, on child BMI^(25)^. Adjusting for physical activity is an essential component when exploring energy balance, due to its significant association with BMI. However, the incorporation of physical activity was restricted to 5–15-year-olds, limiting our ability to generalise these findings to children of all ages. In addition, time-of-day energy intake and BMI associations were assessed within the subsample of 5–15-year-olds *before* controlling for physical activity (see online Supplementary Table S3). The analysis on the subsample of 5–15-year-olds did not replicate the statistically significant associations observed when the analysis was conducted on the full sample of 4–18-year-olds. Therefore, it is difficult to infer the true effect of physical activity on time-of-day energy intake and BMI within the overall sample, given this limitation in the availability of the data on physical activity.

Additional limitations of this study have been noted. The potential risk of type 1 error was not accounted for within the analysis, such as the use of Bonferroni correction, which would consequently increase the risk of type II error^(66)^, or through reducing the significance level to 1 %. Due to the numerous statistical tests performed, it is possible that some significant results may have occurred by chance. However, we have been cautious in our interpretation of the results and have not overstated our findings. In addition, confidence intervals have been presented alongside coefficients and *P* values to allow for transparency of our findings. Additionally, the current analysis did not consider sleep duration as a covariate or individual differences in circadian timing. Since the timing of energy intake and its impact on BMI is largely dependent upon circadian physiology^(34)^, accounting for these factors can provide richer insight into the relationship between individual circadian timing, rather than arbitrary mealtimes and BMI. However, data on sleep duration were only available in the first four waves of the data set and was previously reported to be unavailable for the majority of children^(25)^. In addition, no data were collected on sleep onset and offset; therefore, a calculation of sleep midpoint could not have been used as a proxy for circadian phase. Future research and nationally representative data sets ought to consider capturing these variables within their methods so they can be accounted for within data analysis. Finally, our findings were based on secondary cross-sectional data. We were therefore unable to explore the potential physiological mechanisms of our observations to draw inferences regarding energy intake compensation throughout the day and on subsequent days. There is some evidence, though limited, to suggest that older children^(67)^ and adults^(52,68)^ do not compensate for over-consumption during subsequent mealtimes, thus leading to weight gain. Future research ought to explore the mediating effects of energy compensation as a mechanism for time-of-day energy intake and BMI.

The current study has provided evidence of the importance of energy intake throughout different times of the day on child and adolescent BMI and weight status. Currently, there is a lack of guidance regarding mealtimes, or time-of-day energy intake in a broader sense, and portion sizes for children. This could be due to the conflicting research findings available as well as the questionable reliability of self-reported data. The results of the current study suggest that energy consumption later in the day in the early years may be a risk factor for increased BMI. On the other hand, increased morning consumption may be protective of increased weight amongst older girls. While there are clear national guidelines on recommended daily energy intakes for both children and adults, less guidance is provided on the distribution of energy throughout the day that may help lessen child overweight and obesity. Developing mealtime guidance could promote healthier lifestyles by increasing parental awareness of optimal timing of food intake throughout the day and its potential impact on BMI.

Future advancements in research should consider more objective methods of measuring food intake to eliminate any uncertainty surrounding the reliability of energy intake data. In addition, given the complexity of factors that can potentially impact child BMI, a better understanding of the most important confounders is needed. This will help guide the inclusion of covariates within future analyses to predict the impact of time-of-day or mealtime energy intake on child weight more accurately.

## Supplementary material

For supplementary material accompanying this paper visit https://doi.org/10.1017/S0007114522003014. click here to view supplementary material
