# Supplementary material for: Timing of energy intake and BMI in children: differential impacts by age and sex
Source: Br J Nutr. 2022 Sep 21;130(1):71–82. doi: 10.1017/S0007114522003014 (PMC7614627; doi:10.1017/S0007114522003014)
Supplement: Supplementary file 1 [file S0007114522003014sup001.docx]

**Supplementary material: Additional tables**

|  | | | | | | |
| --- | --- | --- | --- | --- | --- | --- |
| **Supplementary Table S1.** **Relationship between Weight status and timing of Energy Intake** | | | | | | |
|  | | | | | | |
| Dependent var: | Model 1 | Model 2 | Model 3 | | | |
| OW&Obese (1,0) | [95% CI] | [95% CI] | [95% CI] | | | |
| Odds ratios |  |  | Young boy  aged 4-10 | Young girl  aged 4-10 | Older boy  aged 11-18 | Older girl  aged 11-18 |
| Total daily intake | 0.9780 |  |  |  |  |  |
| (kJ×10^3^) | [0.961,0.996] |  |  |  |  |  |
|  | (*p=*0.02) |  |  |  |  |  |
| Intake 05:00-10:59 |  | 0.9588 | 0.9390 | 1.0228 | 0.9848 | 0.9007 |
| (kJ×10^3^) |  | [0.917,1.003] | [0.839,1.051] | [0.905,1.156] | [0.919,1.055] | [0.826,0.982] |
|  |  | (*p=*0.07) | (*p=*0.27) | (*p=*0.72) | (*p=*0.66) | (*p=*0.02) |
| Intake 11:00-14:59 |  | 0.9939 | 1.0143 | 1.0521 | 0.9962 | 0.9608 |
| (kJ×10^3^) |  | [0.966,1.023] | [0.943,1.091] | [0.973,1.138] | [0.953,1.042] | [0.912,1.012] |
|  |  | (*p=*0.67) | (*p=*0.70) | (*p=*0.20) | (*p=*0.87) | (*p=*0.13) |
| Intake 15:00-19:59 |  | 0.9852 | 1.026 | 1.0184 | 0.9702 | 0.9670 |
| (kJ×10^3^) |  | [0.962,1.009] | [0.966,1.089] | [0.949,1.092] | [0.934,1.007] | [0.925,1.011] |
|  |  | (*p=*0.23) | (*p=*0.40) | (*p=*0.61) | (*p=*0.12) | (*p=*0.14) |
| Intake 20:00-04:59 |  | 0.9566 | 1.0270 | 1.0372 | 0.9339 | 0.9508 |
| (kJ×10^3^) |  | [0.926,0.989] | [0.917,1.150] | [0.917,1.173] | [0.891,0.979] | [0.897,1.008] |
|  |  | (*p=*0.009) | (*p=*0.64) | (*p=*0.56) | (*p=*0.004) | (*p=*0.09) |
| Young boy 4-10 yrs | base | base | base | | | |
| Young girl 4-10 yrs | 0.9037 | 0.9033 | 0.7760 | | | |
|  | [0.766,1.066] | [0.766,1.065] | [0.488,1.233] | | | |
|  | (*p=*0.23) | (*p=*0.23) | (*p=*0.28) | | | |
| Older boy 11-18 yrs | 1.4040 | 1.4107 | 1.6960 | | | |
|  | [1.200,1.643] | [1.204,1.653] | [1.131,2.543] | | | |
|  | (*p<*0.000) | (*p<*0.000) | (*p=*0.01) | | | |
| Older girl 11-18 yrs | 1.3296 | 1.3340 | 1.8574 | | | |
|  | [1.141,1.550] | [1.142,1.558] | [1.243,2.777] | | | |
|  | (*p<*0.000) | (*p<*0.000) | (*p=*0.003) | | | |
| Constant | 0.5853 | 0.5761 | 0.4974 | | | |
|  | [0.490,0.700] | [0.480,0.692] | [0.358,0.692] | | | |
|  | (*p<*0.000) | (*p<*0.000) | (*p<*0.000) | | | |
| Ethnicity (5 groups) | ✓ | ✓ | ✓ | | | |
| HH income (4 bands) | ✓ | ✓ | ✓ | | | |
| N | 23,796 | 23,796 | 23,796 | | | |
|  |  |  |  | | | |

Source: National Diet and Nutrition Survey (NDNS) 2008-2019 – authors’ calculations

Notes: Dependent variable is a dichotomous indicator of weight status (overweight or obese vs normal weight). Reported coefficients are odds ratios (OR). 95% confidence intervals in [brackets]; p-values in (parentheses). All specifications include ethnicity (5 categories) and household income tertiles (4 categories including non-reported).

| **Supplementary Table S2.** **Relationship between Weight status, timing of Energy Intake and Physical Activity for 5–15-year-olds** | | | | | | |
| --- | --- | --- | --- | --- | --- | --- |
|  | | | | | | |
| Dependent var: | Model 1 | Model 2 | Model 3 | | | |
| OW&Obese (1,0) | [95% CI] | [95% CI] | [95% CI] | | | |
| Odds ratios |  |  | Young boy  aged 4-10 | Young girl  aged 4-10 | Older boy  aged 11-18 | Older girl  aged 11-18 |
| Total daily intake | 0.9674 |  |  |  |  |  |
| (kJ×10^3^) | [0.935,1.001] |  |  |  |  |  |
|  | (*p=*0.06) |  |  |  |  |  |
| Intake 05:00-10:59 |  | 0.9051 | 0.883 | 0.9898 | 0.9063 | 0.8655 |
| (kJ×10^3^) |  | [0.831,0.986] | [0.735,1.060] | [0.818,1.198] | [0.781,1.051] | [0.733,1.022] |
|  |  | (*p=*0.02) | (*p=*0.18) | (*p=*0.92) | (*p=*0.19) | (*p=*0.09) |
| Intake 11:00-14:59 |  | 1.0083 | 1.0614 | 1.1134 | 0.9913 | 0.9405 |
| (kJ×10^3^) |  | [0.956,1.064] | [0.948,1.188] | [0.978,1.268] | [0.903,1.088] | [0.849,1.042] |
|  |  | (*p=*0.76) | (*p=*0.30) | (*p=*0.10) | (*p=*0.85) | (*p=*0.24) |
| Intake 15:00-19:59 |  | 0.9733 | 0.9739 | 0.9168 | 1.0084 | 0.9643 |
| (kJ×10^3^) |  | [0.930,1.018] | [0.887,1.070] | [0.814,1.033] | [0.935,1.088] | [0.881,1.055] |
|  |  | (*p=*0.24) | (*p=*0.58) | (*p=*0.15) | (*p=*0.83) | (*p=*0.43) |
| Intake 20:00-04:59 |  | 0.9454 | 1.0261 | 0.8709 | 0.918 | 0.9728 |
| (kJ×10^3^) |  | [0.883,1.012] | [0.871,1.209] | [0.705,1.076] | [0.826,1.020] | [0.857,1.104] |
|  |  | (*p=*0.10) | (*p=*0.76) | (*p=*0.20) | (*p=*0.11) | (*p=*0.67) |
| Young boy 4-10 yrs | base | base | base | | | |
| Young girl 4-10 yrs | 0.8099 | 0.8055 | 0.7827 | | | |
|  | [0.625,1.050] | [0.622,1.044] | [0.376,1.630] | | | |
|  | (*p=*0.11) | (*p=*0.10) | (*p=*0.51) | | | |
| Older boy 11-18 yrs | 1.5831 | 1.5803 | 1.6769 | | | |
|  | [1.218,2.058] | [1.213,2.058] | [0.846,3.324] | | | |
|  | (*p=*0.001) | (*p=*0.001) | (*p=*0.14) | | | |
| Older girl 11-18 yrs | 1.2373 | 1.2237 | 1.6469 | | | |
|  | [0.950,1.612] | [0.937,1.597] | [0.826,3.284] | | | |
|  | (*p=*0.12) | (*p=*0.14) | (*p=*0.16) | | | |
| Physical activity:Low | base | base | base | | | |
| Physical activity: | 0.9123 | 0.9129 | 0.9090 | | | |
| Medium | [0.739,1.126] | [0.740,1.126] | [0.736,1.122] | | | |
|  | (*p=*0.39) | (*p=*0.40) | (*p=*0.37) | | | |
| Physical activity: | 0.8661 | 0.8665 | 0.8635 | | | |
| High | [0.660,1.137] | [0.660,1.138] | [0.657,1.134] | | | |
|  | (*p=*0.30) | (*p=*0.30) | (*p=*0.29) | | | |
| Constant | 0.6374 | 0.6371 | 0.5751 | | | |
|  | [0.459,0.885] | [0.457,0.888] | [0.340,0.973] | | | |
|  | (*p=*0.007) | (*p=*0.008) | (*p=*0.04) | | | |
| Ethnicity (5 groups) | ✓ | ✓ | ✓ | | | |
| HH income (4 bands) | ✓ | ✓ | ✓ | | | |
| N | 8,290 | 8,290 | 8,290 | | | |
|  |  |  |  | | | |

Source: National Diet and Nutrition Survey (NDNS) 2014-2019 – authors’ calculations

Notes: See Supplementary Table S1. The physical activity level variable is recorded for waves 6-11 (2014-2019) only, and for children aged 5-15 years. See text for details

| **Supplementary Table S3 Relationship between BMI and Energy Intake for 5–15-year-olds (waves 6-11)** | | | | | | |
| --- | --- | --- | --- | --- | --- | --- |
|  | | | | | | |
| Dependent var: | Model 1 | Model 2 | Model 3 | | | |
| BMI value | [95% CI] | [95% CI] | [95% CI] | | | |
|  |  |  | Young boy  aged 4-10 | Young girl  aged 4-10 | Older boy  aged 11-18 | Older girl  aged 11-18 |
| Total daily intake | -0.0079 |  |  |  |  |  |
| (kJ×10^3^) | [-0.065,0.049] |  |  |  |  |  |
|  | (*p=*0.78) |  |  |  |  |  |
| Intake 05:00-10:59 |  | -0.1300 | -0.1112 | -0.1122 | -0.019 | -0.019 |
| (kJ×10^3^) |  | [-0.278,0.018] | [-0.316,0.094] | [-0.381,0.157] | [-0.331,0.293] | [-0.331,0.293] |
|  |  | (*p=*0.08) | (*p=*0.29) | (*p=*0.41) | (*p=*0.90) | (*p=*0.91) |
| Intake 11:00-14:59 |  | 0.0337 | 0.1091 | 0.1301 | 0.0416 | 0.0416 |
| (kJ×10^3^) |  | [-0.065,0.133] | [-0.032,0.250] | [-0.033,0.293] | [-0.151,0.234] | [-0.151,0.234] |
|  |  | (*p=*0.51) | (*p=*0.13) | (*p=*0.12) | (*p=*0.67) | (*p=*0.67) |
| Intake 15:00-19:59 |  | -0.0001 | 0.0129 | -0.0178 | 0.0234 | 0.0234 |
| (kJ×10^3^) |  | [-0.074,0.074] | [-0.091,0.117] | [-0.152,0.117] | [-0.127,0.174] | [-0.127,0.174] |
|  |  | (*p=*0.99) | (*p=*0.81) | (*p=*0.80) | (*p=*0.76) | (*p=*0.76) |
| Intake 20:00-04:59 |  | 0.0272 | 0.1483 | -0.1126 | 0.0127 | 0.0127 |
| (kJ×10^3^) |  | [-0.095,0.150] | [-0.060,0.357] | [-0.356,0.131] | [-0.202,0.227] | [-0.202,0.227] |
|  |  | (*p=*0.66) | (*p=*0.16) | (*p=*0.36) | (*p=*0.91) | (*p=*0.91) |
| Young boy 4-10 yrs | base | base | base | | | |
| Young girl 4-10 yrs | 0.0689 | 0.0602 | 0.2040 | | | |
|  | [-0.260,0.398] | [-0.269,0.389] | [-0.760,1.168] | | | |
|  | (*p=*0.68) | (*p=*0.72) | (*p=*0.68) | | | |
| Older boy 11-18 yrs | 3.4601 | 3.4271 | 3.4589 | | | |
|  | [2.995,3.926] | [2.962,3.893] | [2.352,4.566] | | | |
|  | (*p<*0.001) | (*p<*0.001) | (*p<*0.001) | | | |
| Older girl 11-18 yrs | 4.3398 | 4.2962 | 5.0684 | | | |
|  | [3.871,4.809] | [3.824,4.768] | [3.905,6.232] | | | |
|  | (*p<*0.001) | (*p<*0.001) | (*p<*0.001) | | | |
| Constant | 17.474 | 17.519 | 17.260 | | | |
|  | [16.97,17.98] | [17.00,18.03] | [16.58,17.94] | | | |
|  | (*p<*0.001) | (*p<*0.001) | (*p<*0.001) | | | |
| Ethnicity (5 groups) | ✓ | ✓ | ✓ | | | |
| HH income (4 bands) | ✓ | ✓ | ✓ | | | |
| N | 8,290 | 8,290 | 8,290 | | | |
| R^2^ | 0.235 | 0.235 | 0.237 | | | |

Source: National Diet and Nutrition Survey (NDNS) 2014-2019 – authors’ calculations

Notes: 95% confidence intervals in [brackets]; p-values in (parentheses).
