# Supplementary material for: Timing of energy intake and BMI in children: differential impacts by age and sex
Source: Br J Nutr. Author manuscript; Available in PMC 2023 Jul 14. (PMC7614627; doi:10.1017/S0007114522003014)
Supplement: Supplementary file 1 [file EMS154241-supplement-Supplementary_Material.pdf]

Supplementary material: Additional tables

Supplementary Table S1. Relationship between Weight status and timing of Energy Intake

Dependent var: OW&Obese (1,0)	Model 1 [95% CI]	Model 2 [95% CI]	Model 3 [95% CI]			
Odds ratios			Young boy aged 4-10	Young girl aged 4-10	Older boy aged 11-18	Older girl aged 11-18
Total daily intake (kJ×10^3)	0.9780 [0.961,0.996] (*p*=0.02)					
Intake 05:00-10:59 (kJ×10^3)		0.9588 [0.917,1.003] (*p*=0.07)	0.9390 [0.839,1.051] (*p*=0.27)	1.0228 [0.905,1.156] (*p*=0.72)	0.9848 [0.919,1.055] (*p*=0.66)	0.9007 [0.826,0.982] (*p*=0.02)
Intake 11:00-14:59 (kJ×10^3)		0.9939 [0.966,1.023] (*p*=0.67)	1.0143 [0.943,1.091] (*p*=0.70)	1.0521 [0.973,1.138] (*p*=0.20)	0.9962 [0.953,1.042] (*p*=0.87)	0.9608 [0.912,1.012] (*p*=0.13)
Intake 15:00-19:59 (kJ×10^3)		0.9852 [0.962,1.009] (*p*=0.23)	1.026 [0.966,1.089] (*p*=0.40)	1.0184 [0.949,1.092] (*p*=0.61)	0.9702 [0.934,1.007] (*p*=0.12)	0.9670 [0.925,1.011] (*p*=0.14)
Intake 20:00-04:59 (kJ×10^3)		0.9566 [0.926,0.989] (*p*=0.009)	1.0270 [0.917,1.150] (*p*=0.64)	1.0372 [0.917,1.173] (*p*=0.56)	0.9339 [0.891,0.979] (*p*=0.004)	0.9508 [0.897,1.008] (*p*=0.09)
Young boy 4-10 yrs	base	base	base			
Young girl 4-10 yrs	0.9037 [0.766,1.066] (*p*=0.23)	0.9033 [0.766,1.065] (*p*=0.23)	0.7760 [0.488,1.233] (*p*=0.28)			
Older boy 11-18 yrs	1.4040 [1.200,1.643] (*p*<0.000)	1.4107 [1.204,1.653] (*p*<0.000)	1.6960 [1.131,2.543] (*p*=0.01)			
Older girl 11-18 yrs	1.3296 [1.141,1.550] (*p*<0.000)	1.3340 [1.142,1.558] (*p*<0.000)	1.8574 [1.243,2.777] (*p*=0.003)			

Constant	0.5853<br>[0.490,0.700]<br>(p<0.000)	0.5761<br>[0.480,0.692]<br>(p<0.000)	0.4974<br>[0.358,0.692]<br>(p<0.000)
Ethnicity (5 groups)	✓	✓	✓
HH income (4 bands)	✓	✓	✓
N	23,796	23,796	23,796

Source: National Diet and Nutrition Survey (NDNS) 2008-2019 – authors' calculations

Notes: Dependent variable is a dichotomous indicator of weight status (overweight or obese vs normal weight). Reported coefficients are odds ratios (OR). 95% confidence intervals in [brackets]; p-values in (parentheses). All specifications include ethnicity (5 categories) and household income tertiles (4 categories including non-reported).

Supplementary Table S2. Relationship between Weight status, timing of Energy Intake and Physical Activity for 5–15-year-olds

Dependent var: OW&Obese (1,0)	Model 1 [95% CI]	Model 2 [95% CI]	Model 3 [95% CI]			
Odds ratios			Young boy aged 4-10	Young girl aged 4-10	Older boy aged 11-18	Older girl aged 11-18
Total daily intake ($kJ\times10^3$)	0.9674 [0.935,1.001] (p=0.06)					
Intake 05:00-10:59 ($kJ\times10^3$)		0.9051 [0.831,0.986] (p=0.02)	0.883 [0.735,1.060] (p=0.18)	0.9898 [0.818,1.198] (p=0.92)	0.9063 [0.781,1.051] (p=0.19)	0.8655 [0.733,1.022] (p=0.09)
Intake 11:00-14:59 ($kJ\times10^3$)		1.0083 [0.956,1.064] (p=0.76)	1.0614 [0.948,1.188] (p=0.30)	1.1134 [0.978,1.268] (p=0.10)	0.9913 [0.903,1.088] (p=0.85)	0.9405 [0.849,1.042] (p=0.24)
Intake 15:00-19:59 ($kJ\times10^3$)		0.9733 [0.930,1.018] (p=0.24)	0.9739 [0.887,1.070] (p=0.58)	0.9168 [0.814,1.033] (p=0.15)	1.0084 [0.935,1.088] (p=0.83)	0.9643 [0.881,1.055] (p=0.43)
Intake 20:00-04:59 ($kJ\times10^3$)		0.9454 [0.883,1.012] (p=0.10)	1.0261 [0.871,1.209] (p=0.76)	0.8709 [0.705,1.076] (p=0.20)	0.918 [0.826,1.020] (p=0.11)	0.9728 [0.857,1.104] (p=0.67)
Young boy 4-10 yrs	base	base	base			
Young girl 4-10 yrs	0.8099 [0.625,1.050] (p=0.11)	0.8055 [0.622,1.044] (p=0.10)	0.7827 [0.376,1.630] (p=0.51)			
Older boy 11-18 yrs	1.5831 [1.218,2.058] (p=0.001)	1.5803 [1.213,2.058] (p=0.001)	1.6769 [0.846,3.324] (p=0.14)			
Older girl 11-18 yrs	1.2373 [0.950,1.612] (p=0.12)	1.2237 [0.937,1.597] (p=0.14)	1.6469 [0.826,3.284] (p=0.16)			
Physical activity:Low	base	base	base			

Physical activity:	0.9123	0.9129	0.9090
Medium	[0.739,1.126]	[0.740,1.126]	[0.736,1.122]
	(*p*=0.39)	(*p*=0.40)	(*p*=0.37)
Physical activity:	0.8661	0.8665	0.8635
High	[0.660,1.137]	[0.660,1.138]	[0.657,1.134]
	(*p*=0.30)	(*p*=0.30)	(*p*=0.29)
Constant	0.6374	0.6371	0.5751
	[0.459,0.885]	[0.457,0.888]	[0.340,0.973]
	(*p*=0.007)	(*p*=0.008)	(*p*=0.04)
Ethnicity (5 groups)	✓	✓	✓
HH income (4 bands)	✓	✓	✓
N	8,290	8,290	8,290

Source: National Diet and Nutrition Survey (NDNS) 2014-2019 – authors' calculations

Notes: See Supplementary Table S1. The physical activity level variable is recorded for waves 6-11 (2014-2019) only, and for children aged 5-15 years. See text for details

Supplementary Table S3 Relationship between BMI and Energy Intake for 5–15-year-olds (waves 6-11)

Dependent var: BMI value	Model 1 [95% CI]	Model 2 [95% CI]	Model 3 [95% CI]			
			Young boy aged 4-10	Young girl aged 4-10	Older boy aged 11-18	Older girl aged 11-18
Total daily intake (kJ×10^3)	-0.0079 [-0.065,0.049] (*p*=0.78)					
Intake 05:00-10:59 (kJ×10^3)		-0.1300 [-0.278,0.018] (*p*=0.08)	-0.1112 [-0.316,0.094] (*p*=0.29)	-0.1122 [-0.381,0.157] (*p*=0.41)	-0.019 [-0.331,0.293] (*p*=0.90)	-0.019 [-0.331,0.293] (*p*=0.91)
Intake 11:00-14:59 (kJ×10^3)		0.0337 [-0.065,0.133] (*p*=0.51)	0.1091 [-0.032,0.250] (*p*=0.13)	0.1301 [-0.033,0.293] (*p*=0.12)	0.0416 [-0.151,0.234] (*p*=0.67)	0.0416 [-0.151,0.234] (*p*=0.67)
Intake 15:00-19:59 (kJ×10^3)		-0.0001 [-0.074,0.074] (*p*=0.99)	0.0129 [-0.091,0.117] (*p*=0.81)	-0.0178 [-0.152,0.117] (*p*=0.80)	0.0234 [-0.127,0.174] (*p*=0.76)	0.0234 [-0.127,0.174] (*p*=0.76)
Intake 20:00-04:59 (kJ×10^3)		0.0272 [-0.095,0.150] (*p*=0.66)	0.1483 [-0.060,0.357] (*p*=0.16)	-0.1126 [-0.356,0.131] (*p*=0.36)	0.0127 [-0.202,0.227] (*p*=0.91)	0.0127 [-0.202,0.227] (*p*=0.91)
Young boy 4-10 yrs	base	base	base			
Young girl 4-10 yrs	0.0689 [-0.260,0.398] (*p*=0.68)	0.0602 [-0.269,0.389] (*p*=0.72)	0.2040 [-0.760,1.168] (*p*=0.68)			
Older boy 11-18 yrs	3.4601 [2.995,3.926] (*p*<0.001)	3.4271 [2.962,3.893] (*p*<0.001)	3.4589 [2.352,4.566] (*p*<0.001)			
Older girl 11-18 yrs	4.3398 [3.871,4.809] (*p*<0.001)	4.2962 [3.824,4.768] (*p*<0.001)	5.0684 [3.905,6.232] (*p*<0.001)			
Constant	17.474 [16.97,17.98]	17.519 [17.00,18.03]	17.260 [16.58,17.94]			

	(p<0.001)	(p<0.001)	(p<0.001)
Ethnicity (5 groups)	✓	✓	✓
HH income (4 bands)	✓	✓	✓
N	8,290	8,290	8,290
R^2	0.235	0.235	0.237

Source: National Diet and Nutrition Survey (NDNS) 2014-2019 – authors' calculations

Notes: 95% confidence intervals in [brackets]; p-values in (parentheses).
